# Cytogenetic methods for detection of oxidative stress and evaluation of antioxidant therapy in hepatitis C infection

**Published:** 2011-06-01

**Authors:** Ingrid Emerit

**Affiliations:** 1 Paris VI University and National Center of Scientific Research, Paris, France

**Keywords:** Hepatitis C virus, Oxidative stress, Cytogenetic

## Abstract

The plasma of patients with hepatitis C contains chromosome-damaging substances, the so-called "clastogenic factors" (CFs), as this is the case for other chronic inflammatory diseases and after radiation exposure. These endogenous clastogens, formed as a consequence of increased superoxide production by inflammatory cells, can be detected with cytogenetic methods, as they are used for exogenous clastogens. The long-lived, autosustained DNA-damaging effects of CFs are risk factors for the development of cancer and leukemia. In hepatitis C, the highest clastogenic scores has been observed in patients with hepatocellular carcinoma. In agreement with the link to inflammation, clastogenic score are correlated with necro-inflammatory scores in liver biopsies. Antioxidant therapy with a powerful superoxide scavenger resulted in normalization of clastogenic scores and significant decreases in aminotransferase levels, but did not influence the virus load. Preliminary results of our study on a limited number of patients suggest that pre-treatment with antioxidants may improve the outcome of interferon/ribavirin treatment. A comparison of a three-month treatment with either interferon alone or the antioxidant alone, yielded similar results for reduction of ALT levels, but only complete normalization of clastogenic scores for the antioxidant. Further studies have to be conducted to see whether a combination of an antiviral agent with an appropriate antioxidant would allow to reduce interferon and its side effects.Combination of antioxidants with IFN/RIBA was also reported by other authors with discordant results. The CF-test can be useful in clinical trials for the choice of the appropriate antioxidant.

## 1. Introduction

Oxidative stress is involved in chronic hepatitis C, and efforts have been made to influence the disease process with antioxidants [1][2][3]. Oxidative damage has been documented in lipids, proteins and DNA. Increased oxyradical production could be detected on liver biopsies by direct measurements with spin trapping [4]. High resting levels of superoxide anion in the whole blood of patients were detected with chemiluminescence techniques[5]. We have reported previously that clastogenic (i.e., chromosome damaging) substances, the so-called clastogenic factors (CFs), are present in the plasma of patients with hepatitis C [6], as this is the case for a variety of other pathological conditions accompanied by oxidative stress. These include irradiation exposure, chronic inflammatory diseases, HIV infection, ischemia reperfusion injury, the hereditary chromosomal instability syndromes and others [7]. The formation, as well as the chromosome damaging effects of CFs, is mediated by the superoxide anion radical since they are regularly inhibited by superoxide dismutase (SOD) or other superoxide scavengers. For this reason, the term "superoxide-mediated clastogenesis" has been proposed [8]. Superoxide is not a direct DNA-damaging agent, but an initiator of a series of events leading to the formation of clastogenic materials. Biochemical analysis has identified lipid peroxidation products, arachidonic acid metabolites, nucleotides of inosine and cytokines, in particular tumor necrosis factor (TNF) alpha, as the clastogenic and also superoxide stimulating components of CFs. Due to their chromosome-damaging properties, these substances can be detected with classical cytogenetic methods.

## 2. Formation and action of CF

When cell cultures are exposed to superoxide-generating systems, such as a xanthine-xanthine oxidase reaction or a respiratory burst stimulated by a tumor promoter, mitotic cells show chromosomal breakage, and the supernatant of these cultures induces breakage, when transferred to other cell cultures. This could be consistently prevented by SOD. Exposure of cell-free culture medium or serum does not result in clastogenic activity, indicating that CFs are cellular products. After resuspension of the cells in fresh medium, they continue to release CFs in absence of the superoxide generating system. Similar to the events observed in cell culture under experimental conditions, CF formation can occur in the organisms during inflammatory diseases via a respiratory burst by competent cells. After activation of phospholipase A, arachidonic acid (AA)-derived eicosanoids are released from cellular membranes and further degraded to the breakdown product 4-hydroxynonenal (HNE), which is highly clastogenic at 0.1 µM concentration. This pathway of CF formation can be blocked not only by dismutation of the initiating superoxide by SOD, but also by inhibitors of the AA cascade. In culture systems, the formation of HNE needs a minimum of 18 hours, without further increase after 24 hours. SOD or other inhibitors of CF formation are most efficient, if added to the cultures during the first 24 hours of the cultivation period, i.e., before accumulation of HNE. This aldehyde has no superoxide generating properties, but is responsible for DNA damage by inactivation of functional SH groups of DNA polymerases and adduct formation with cellular thiols.

Superoxide generation via a respiratory burst also activates another enzyme-protein kinase C. This leads to release of TNF-α, the clastogenic properties of which could be confirmed by the study of the commercial product. The presence of TNF in CFs leads to further superoxide generation and amplifies the process. Chronic inflammation is also accompanied by increases in adenosine deaminise and xanthine oxidase. This leads to nucleotide pool imbalances with formation of inosine tri- and di-phosphate, not produced in the organism under normal conditions. These nucleotides are not only clastogenic, but have also superoxide stimulating properties, as demonstrated with chemiluminescence or the cytochrome C reduction assay. Amplification of CF formation is the consequence, as mentioned above for TNF-α. In addition, ITP raises intracellular calcium levels, activates lysosomal enzymes and induces chromosomal damage through the action of nucleases. Competition between ITP and ATP-binding sites of DNA-polymerases may also affect DNA repair. We may deduce that CF formation as well as CF action is multifactorial and multistep processes, responsible for an autosustained DNA damaging process [7].

As in other chronic inflammatory diseases, these mechanisms of CF formation and action are implicated in patients with hepatitis C. Monocytes of HCV-infected persons are in an activated state and represent a source of superoxide for CF formation[9]. The Kupffer cells of the liver represent the largest population of macrophages in the human body. They release various mediators including superoxide, eiosanoids, and cytokines. TNF-α is produced in excess in chronic hepatitis [10]. Increased levels of xanthine oxidase, coupled with an increase in the substrates xanthine and xanthine oxidase were noted in virus-infected tissues [11]. The xanthine oxidase reaction may therefore be another source of superoxide in hepatitis C. Also the enzyme adenosine deaminise was found to be increased in patients with viral hepatitis [12] leading to ITP formation, as described above. Since it was shown that lipid peroxidation products are increased in plasma [13] and liver of patients [14], the clastogenic degradation product HNE should be present also. The fact that CFs of hepatitis C patients were detected in the same low molecular weight fraction as CFs of other origin, as well as the anticlastogenic effects of SOD are arguments for similarities in their composition. Clastogenic action of HCV can be excluded, given the pore size of the ultrafiltration system retentive for viruses.

## 3. Measurement of clastogenic activity: The CF test

CFs are endogenous clastogens, and the methods for their detection follow the protocols established for the study of chromosomal breakage after exposure to exogenous clastogens. Regular blood cultures set up with 0.5 mL of blood from a healthy donor are exposed to plasma or plasma ultrafiltrates from patients. Ultrafiltrates are preferable, since the ultrafiltration step eliminates all high molecular weight materials, which might disturb culture growth in case of blood group incompatibilities. Also residual cells in patients' plasma are retained by the filter. Since the clastogenic activity is in the small molecular weight fraction, the plasma is filtered through Millipore ultrafiltration filters with a cutoff at 30,000 Daltons. The ultrafiltrates are unstable at room temperature and loose also their activity in the refrigerator overnight. Frozen, they can be conserved over years. In order to avoid repeated freezing and thawing, aliquots of the samples should be prepared. The clastogenic activity is not lost during lyophilization, if done rapidly in small aliquots.

The culture medium has to be poor in free radical scavengers. TCM 199 or RPMI 1640 mL (5 mL per culture tube) are recommended, while RPMI 1629 or Ham's FT are not convenient because of their high L-cysteine content. The medium is supplemented with fetal calf serum (1 mL per culture). It should have a yellow aspect indicating that there was no hemolysis during serum preparation. Otherwise SOD from lysed erythrocytes can be present at anticlastogenic quantities. Bovine serum may be rich in vitamin E and inhibit clastogenesis related to lipid peroxidation. The usual quantity of ultrafiltrate varies between 100 and 200 µL for a total of 5 mL medium plus 1 mL serum per culture. Higher doses may have cytotoxic effects resulting in "false negative results." The CF test can be done only on well proliferating cultures, and therefore the test has to be repeated with lower doses. On the other hand, if 100 µL do not induce breakage, the culture has to be repeated with a higher dose. If possible, both quantities should be tested simultaneously. Addition of CF at the beginning of the cultivation period yields the highest aberration rates. This is explained by the multifactorial, multistep process of CF action with amplification of the initial clastogenic activity.

Lymphocyte proliferation is stimulated by the addition of phytohemagglutinin. After 48 or 72 hr of incubation at 37 °C, the mitoses are arrested in metaphase by the addition of colchicine, 2 hr before harvesting. The mitosis rate is in general higher at 72 hr, but 48 hr cultures are preferable, if the nature of chromosomal aberrations and not only breaks are evaluated. Microscopic slides are prepared according to classical cytogenetic procedures. The tubes are centrifuged at low speed (800-1000 t) for 10 min. The pellet is suspended in 10 mL of hypotonic KCl (0.28%-0.4%) for 20 min at 37 °C. After centrifugation, the cells are fixed in alcohol-acetic acid (3:1). Two to three changes are necessary until the supernatant does no longer show traces of hemoglobin. After the last change, the pellet is suspended in several drops of ice-cold glacial acetic acid, spread on cold wet slides and dried at room temperature or with a hair dryer. Good spreading is only obtained on absolutely clean slides, on which the drop is not retracting. The chromosomes are stained with Giemsa, and 50 well-spread metaphase plates are examined on coded slides for the presence of chromatid type aberrations (chromatid and isochromatid breaks, telomeric extrusions or acentric fragments). Gaps are not considered as aberrations. Chromosome type aberrations such as rings, dicentrics or other structurally rearranged chromosomes are rarely observed. This is typical for CF-induced chromosomal breakage and due to the multifactorial process of break induction.

A series of samples is tested the same day on the cultures set up with the same donor blood. One or two additional cultures are set up without ultrafiltrate as controls. The background level of aberrations in the control is deduced from the aberration rate in the ultrafiltrate-treated cultures. The difference of the two values is called the adjusted clastogenic score (ACS). The background level of the control culture should not exceed 2%. Donors exhibiting increased chromosomal breakage for other reasons, i.e. exposure to x-ray procedures, are not suitable for the CF-test. Ultrafiltrates prepared from blood of healthy adults are not clastogenic, as ascertained in our laboratory by the study of 100 samples. Only five of these samples induced three additional aberrations for 100 mitoses studied (ACS + 3, or 6%). The others induced one or two aberrations or no aberrations at all. According to these data, ACS + 4 (8%) or higher are CF+. The Mean ± SD ACS for the 100 studied samples was 0.8% ± 1.0%. In another series of out-patients consulting for various other health problems, (called "sick controls"), the Mean ± SD value was higher (3.3% ± 2.1%).

Since chromosome type aberrations are rare, the observer may score only open breaks of one or both chromatids or acentric fragments. They do not need cytogenetic training therefore. The study of sister chromatid exchanges may be proposed, instead. However, comparative studies have shown that break events are 3-5 times more frequent in CF-treated cultures compared to controls, while SCEs, though regularly increased, are generally not even doubled. Fibroblasts, endothelial or mesothelial cells of human origin or from other species may be used for the CF test instead of blood, but the breakage rates are lower. This is due to the presence of monocytes in the blood cultures, which are responsible for the above mentioned amplification process. The dividing lymphocytes are the indicators of the damage. For the same reason, cultures set up with pure lymphocytes are not appropriate. CFs do not induce lesions in isolated DNA. They can be studied only on cellular systems because of the indirect action mechanism of break induction.

The concentration of each of the clastogenic components may not reach detectable levels in biochemical assays. The clastogenic action is the result of the synergistic action of all CF components. Therefore the cytogenetic assay is more sensitive. Since the clastogenic activity in patients' plasma induces also chromosomal breakage in their own cells, cytogenetic studies can be done also on patients' blood cultures. However, these studies have to be conducted on freshly drawn blood, while the study of CF in the plasma can be done on frozen ultrafiltrates after accumulation of samples.

## 4. Results obtained with the CF-test in patients with chronic hepatitis C

In confirmation of our previous findings in other chronic inflammatory diseases, patients with hepatitis C exhibit increased chromosomal breakage, and their plasma exerts clastogenic effects in blood cultures of healthy donors. In a first series of 20 patients [6], the Mean ± SD ACS was 9.30% ± 2.85%, while that of the 20 healthy controls was 1.1% ± 2.4% (p < 0.001). No clastogenic effects were produced, if the test cultures received simultaneously SOD. In 19 patients, the ACS was 8% or higher, i.e. 95% of patients were CF+ (range: 8%-14%). The patients were treated with interferon (IFN-α, three times a week at a dose of 5 MU). After three months, only three of 20 studied patients were still CF+. The Mean ± SD ACS was reduced to 3.8% ± 3.3%. This is still increased compared to healthy controls, but near the values observed in "sick controls" (3.3% ± 2.1%). After discontinuation of the treatment at 12 months, the ACS values were in the same range and remained constant during the six months of follow-up. Alanine aminotransferase (ALT) levels were increased in all patients and decreased progressively under treatment from a Mean ± SD level of 100 ± 70 IU/L before the treatment to 80 ± 80 IU/Lt three months, 70 ± 43 IU/L at 12 months. ALT remained stable until the end of the six months of follow-up (70 ± 4 2 IU/L). There was no correlation between ALT level and ACS for individual results. However, when groups were formed, the group of nine patients with normalized ACS (3.11% ± 1.4%) had also normalized ALT levels (33.1 ± 8.31 IU/L). In agreement with the link to inflammation, ACS was correlated with necroinflammatory scores in liver biopsies. However, viral load and ACS were not correlated. If persistence of normal results for ALT and absence of detectable replicating virus six months after discontinuation of the treatment is conventionally defined as a "sustained response," five of the 20 patients treated with INF-α were responders (SR), 11 were non-responders (NR) and four developed relapse (RR). The mean ACS scores were lower in SR than in NR and RR.

Another series of patients received ribavirin and pegylated interferon alpha 2B (PEG-IFN/RIBA) as a conventional antiviral treatment over a period of 12 months and were followed for six months [15]. This treatment was started after a pre-treatment with an antioxidant during three months. Since we knew from our in vitro results with SOD that the clastogenic effect of CF is related to superoxide production, we choose a product known for its strong superoxide-scavenging activity, the antioxidant Biofactor AOB (AOA Co. Kobe, Japan). AOB is a processed grain food, extracted from various plants according to a patented procedure, during which fermentation with Aspergillus orizae plays a role for the bioavailability of the active compounds, in particular flavonoids and phenols. The flavonoid rutin is present at high concentrations (33 mg/100 g). Other major components are the isoflavons daidz(e)in and genest(e)in. AOB contains also vitamins and various trace elements with antioxidant properties, but at very low concentrations. AOB was compared to a placebo, extracted from other plants, but of similar aspect and very low superoxide-scavenging capacity. For the same inhibitory effect on superoxide production by a xanthine-xanthine oxidase reaction, 4.5 mg/mL of the placebo were needed for only 0.0113 mg/mL AOB. In agreement herewith, the placebo did not significantly reduce the ACS during the three-month treatment. On the opposite, AOB was highly anticlastogenic and reduced ACS from a Mean ± SD of 12.4% ± 1.8% to 3.7% ± 3.3% (p < 0.001). ACS further decreased during the following months to normal values for healthy adults (1.8% ± 1.6%) despite discontinuation of AOB. Significant decreases were also observed for aminotransferase levels in the AOB group, but not in the placebo group. Mean values for HCV-RNA, however, remained unchanged in both groups.

When the antiviral treatment started, the AOB-pre-treated group had normalized ACS (1.8% ± 1.6%), while the placebo pre-treated group had highly increased ACS (10.6% ± 4.6%; p < 0.0001). Thus both groups differed with respect to oxidative stress and chromosomal breakage. Also, the mean aminotransferase levels were lower compared to the placebo-pre-treated group (104.8 IU for ALT and 44.0 IU for AST compared to 147.0 and 74.6 IU, respectively). Six months after discontinuation of the 12-month antiviral therapy, a sustained response was observed in five of nine patients pre-treated with AOB, while the seven patients pre-treated with placebo were all non-responders. One may ask, whether the more favorable outcome of PEG-IFN/RIBA treatment for the group pre-treated with AOB was related to the better conditions at the start due to the antioxidant therapy.

## 5. Clastogenic factors and hepatocellular carcinoma

When the HCV-infected patients were compared among each other, the mean values and percentages of CF-positive samples were higher for patients with cirrhosis and hepatocellular carcinoma compared to patients without these complications (11.1% ± 2.7%, 11.1% ± 4.0% and 0.8% ± 4.4%, respectively) [16]. The differences are not statistically significant, probably because of the small number of participants and the high standard deviation. If the number of CF + patients was compared instead of mean ACS, 34 of the 40 hepatitis C patients without complications were CF + (85%) compared to 16 of 17 patients (94%) with liver cirrhosis and 16 of 17 patients (94%) with hepatocellular carcinoma. For 10 of the 17 hepatoma samples, the culture failed with the usual dose of 200 µL ultrafiltrate and had to be repeated with 100 µL. Despite the reduced volume of clastogen, the ACS were highest in these 10 cultures (12.6% ± 4.2%), while the ACS in the seven other cultures exposed to 200 µL were similar to those of patients with liver cirrhosis (11.1% ± 4.0% and 11.1% ± 2.7%, respectively). The patients with hepatocellular carcinoma were compared to a group of patients with liver metastases from other malignancies. Of whom 45% were CF + with a Mean ± SD ACS of 8.7 ± 4.3, tested with the usual quantity of 200 µL. If these patients were tested with 100 µL in comparison to the hepatoma patients, the differences in ACS were significant (3.6% ± 3.2% and 12.6% ± 4.2%, respectively). Among the 11 patients with metastatic liver disease, only one was CF + in contrast to all 10 patients with hepatoma. High clastogenic activity observed with 100 µL plasma ultrafiltrate after culture failure with the usual dose of 200 µL suggests HCV-positive hepatoma.

## 6. Clastogenic factors and carcinogenesis

Radiobiologists introduced the term "clastogenic plasma factor" in the early 1970s. They already proposed that they were responsible for late effects of radiation such as cancer and leukemia in irradiated subjects [17]. In more recent years, the generation of biologically active, diffusible products by exposure to alpha particles has been considered as a possible mechanism for radon-induced carcinogenesis in the respiratory tract [18]. The strongest evidence for the involvement of chromosome mutation in carcinogenesis came from the cytogenetic studies of the so-called "chromosome breakage syndromes," rare hereditary disorders associated with the highest cancer incidence in humans [19]. Previous studies of our laboratory indicate that CFs are regularly produced in a variety of pathologic conditions accompanied by chronic inflammation, oxidative stress and CF formation [20]. In Crohn's disease, the risk of colorectal cancer is 20 times greater than in the general population [21]. Also, among the connective tissue diseases, the cancer risk is considered to be increased. In patients with progressive systemic sclerosis, lung and esophagus cancer occur on the basis of sclerotic fibrosis [22]. In association with dermatomyositis, a wide variety of tumors has been observed [23], while the malignancies observed in Sjogrens's syndrome are almost exclusively lymphoreticular in type [24]. This seems to be the case also in patients with rheumatoid arthritis, periarteritis nodosa and lupus erythematosus. The data on lupus erythematosus are summarized in a Finnish study [25], confirming previous reports of increased cancer incidence in these patients. A correlation between increased chromosomal breakage, the presence of CFs in the plasma, and a high frequency of tumors was also documented in an animal model for lupus erythematosus in the New Zealand Black (NZB) mouse [26]. By selective matings according to chromosomal breakage frequencies in bone marrow cells, it was possible to develop two NZB sublines-a high-breakage (HB) and a low-breakage (LB) line. At the age of 18 months, the frequency of tumors was four times higher in HB than in LB mice. There was a significant difference for CF activity in the plasma and for spontaneous superoxide production by peritoneal macrophages from these mice. The HB and LB lines differed also significantly in retrovirus production. It has been shown that extracellular superoxide generation activates proto-oncogenes and induces mutations at tumor suppressor loci [27]. The genomic instability increases the chance that mutation(s) required for neoplastic transformation would occur [1]. Chromosomal breakage visible under the microscope is an indicator for events occurring in DNA. It demonstrates that the antioxidant defences and the DNA repair system are overwhelmed. As mentioned above, certain components of CF have not only superoxide-stimulating properties, but are also interfering with DNA repair enzymes. The pathway of carcinogenesis via CF formation appears to be of particular interest because superoxide scavengers can be used as anticarcinogens. CF-induced DNA damage may be responsible also for the high risk of liver cancer in patients with hepatitis C. The detection of CFs indicates that the chromosomal damage in patients' cells is not transient, but that the person will be exposed to clastogenic effects as long as the vicious circle of CF formation via superoxide and stimulation of more superoxide by CF is not interrupted.

## 7. Conclusion

The CF-test is a sensitive assay for the detection of oxidative stress in hepatitis C. At the same time, this test demonstrates the occurrence of damage to DNA, a risk factor for the development of cirrhosis and hepatocellular carcinoma. Clastogenic effects correlate with necroinflammatory activity in the liver and can be an indicator for the need of a liver biopsy. In clinical trials, the test can be useful for the choice of an antioxidant for complementary treatment. Larger series of patients have to be studied combining antioxidant with antiviral treatment.
